# Online Cancer Services: Types of Services Offered and Associated Health Outcomes

**DOI:** 10.2196/jmir.7.3.e35

**Published:** 2005-07-01

**Authors:** Gary C Doolittle, Ashley Spaulding

**Affiliations:** ^1^Department of Internal MedicineDivision of Clinical OncologyUniversity of Kansas Medical CenterKansas CityKSUSA

**Keywords:** Electronic mail, health outcomes, Internet, online cancer services, online support group

## Abstract

There are advantages and disadvantages associated with utilization of online health services among individuals living with cancer. Accessing accurate, reliable health-related information online gives patients the power to enhance their understanding of information they obtain from their health care providers. However, online health information can often be confusing for patients to interpret, and it can sometimes be conflicting or incorrect. Based on a framework by Eysenbach, the following paper discusses various types of cancer services that are available online, and it addresses both positive and negative health outcomes that have been linked to utilizing such services.

## Introduction

According to a recent systematic review and meta-analysis of cross-sectional surveys from various health care institutions, approximately 39% of individuals living with cancer use the Internet [1], indicating that online health services have become an important information source for many patients. Such services are prevalent and are varied in their scope, ranging from electronic mail communication with health care providers, friends, family members, and other patients to virtual support groups for patients and caregivers. Due to the vast availability of online health services today, as well as increased patient interest in knowing about the diagnosis, treatment, and follow-up of cancer [2], continued investigation into their impact on the health outcomes of patients with cancer is imperative.

## Cancer Services Offered Online

**Figure 1:**
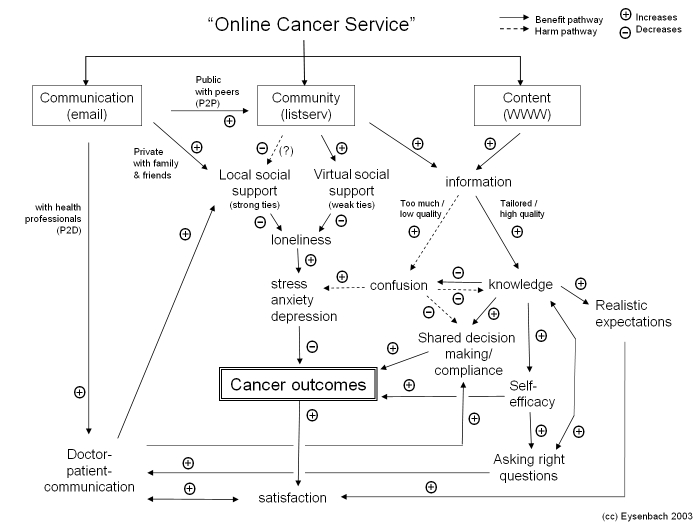
Eysenbach's framework of online cancer services and their possible relationships to health outcomes (reproduced with permission from [1], © Lippincott Williams & Wilkins)

The World Wide Web is the first thing that comes to mind for many people when they hear the phrase “online health services.” However, as noted by Eysenbach [1], while the Web is certainly a common source of health-related information for patients, caregivers, and health care providers alike, online health services encompass quite a bit more than the Web or Internet alone, and a framework of outcomes should be best discussed under the headings “Communication,” “Content,” and “Community” (Figure 1).

### Communication

The primary channel for promoting cancer prevention is communication [3]. Effective health communication—either delivered via a health care provider or via online health services—can have a profound effect on the lives of patients living with cancer. For example, constructive communication about health-related issues can promote prevention of cancer, inform detection and diagnosis of cancer, direct decisions surrounding options for cancer treatment, enhance the ability of cancer survivors to cope with life after the disease, and encourage the best possible end-of-life care [3,4].

Interestingly, as noted by Eysenbach [1], despite the astounding—and frequently overwhelming—amount of information available on the World Wide Web, Internet users cite electronic mail as the number one reason for being online [5]. In his paper, Eysenbach goes on to note that, despite patients' interest in communicating with their physicians via email, less than 10% of patients in the United States have done so [6] because physicians have not yet adopted email as a regular method of communicating with patients out of fear of an increased demand on their time [1]. Furthermore, some physicians have expressed concern about being able to incorporate email communication with patients into their daily routine, about responding to patient email inquiries in a timely manner, and about dealing with content that could be potentially inappropriate or urgent in patient email messages [1].

Eysenbach stresses that communicating with physicians is not the only health-related use patients find for email. Given that family and friends are one of the most frequently cited sources of information for patients with cancer [7], many patients also utilize email to communicate with these family members and friends about issues related to their disease. Additionally, email has the potential to create a sort of virtual support group for patients living with cancer. Often, family and friends of individuals with cancer connect their loved ones with others in their lives who have also been affected by the disease.

In addition to email communication, the general public now has access to real-time assistance through applications such as *LiveHelp*. *LiveHelp* is an instant messaging service initiated as a pilot project in the year 2000 by the Office of Communications at the National Cancer Institute's (NCI's) Cancer Information Service (CIS) [8]. The goal of the service is to assist users with navigating the NCI website in an efficient, confidential manner. Not long after the service was first introduced, almost 4000 *LiveHelp* user sessions transpired from April through December 2001, which is an average of 444 user sessions per month [8]. In general, user feedback and comments about the service have been overwhelmingly positive [8].

*LiveHelp* is available to any individual who has access to the Internet, as no additional computer software or hardware is needed to utilize the service. While public response to the service has generally been positive, the NCI's CIS also has information specialists who are able to answer cancer-related questions via telephone for those who prefer this method of communication and for individuals who cannot readily access the Internet. Through the CIS toll-free telephone number (1-800-4-CANCER), callers have the ability to speak with knowledgeable information specialists who have a lot of experience explaining medical information in easily comprehensible terms [9], which is a particularly important consideration given the vast proliferation of often confusing medical information currently available online.

### Content

Although electronic mail may be the number one *reason* cited by users for being online, it has been argued that the most common *use* of the Internet is information seeking related to medical assistance [10]; however, it has recently been shown that health-related searches actually constitute only 4.5% of all searches in general search engines [11]. Accessing medical information about specific health-related issues on the Internet has been shown to have positive health outcomes for patients with breast cancer in particular [12]. While accessing medical information on the Internet may result in certain positive health outcomes for some patients, there are inherent disadvantages as well to accessing this type of online health service.

One of the biggest challenges when accessing medical information online is the potential for the information to be inaccurate as the Internet contains a staggering amount of medical misinformation [13]. Health care professionals have expressed other concerns about content on the World Wide Web as well. Using a structured search experiment, researchers assessed the accessibility of health information on breast cancer, depression, obesity, and childhood asthma using 14 Internet search engines. Amazingly, less than 25% of the search engines' first pages of links connected the user with relevant, usable content [14]. Additionally, 100% of the English websites and 86% of the Spanish websites required at least a high school reading level [14]. Authors of studies such as these have argued that health-related content found online can often be hard to access and, if found, can be difficult to comprehend.

### Community

The last group of online health services reviewed by Eysenbach [1] is virtual support groups. Similar in nature to traditional, face-to-face support groups, online groups offer patients the opportunity to gain support from someone who has experienced their same illness or from someone who has been through similar treatment [15]. Such groups can be particularly beneficial to cancer patients who may be experiencing pain and/or additional side effects from their disease or treatment as they can participate in an online support group without having to physically travel, provided that they have access to the Internet at home. In addition to the convenience and comfort of participating in an online support group from home, patients have the ability to access social support online anytime day or night. Unlike traditional face-to-face support groups that are scheduled at a particular time in a specific location, patients can participate in online support groups at a time that best meets their needs. Furthermore, provided that they have readily available Internet access, they can participate in online groups instantly. Once again, this is especially beneficial to patients with cancer, whose illness may keep them awake during the night, because the ability to instantly connect with people who have had similar experiences may serve to alleviate some of the anxiety surrounding their illness [15]. Echoing the findings of positive health outcomes for breast cancer patients who access medical information on the Internet, researchers found that women with breast cancer who participated in an online support group also achieved positive health outcomes [16].

## Health Outcomes Associated with Utilization of Online Cancer Services

Researchers' knowledge about factors that facilitate or impede communication, such as access to, sources of, and trust of cancer-related information, is limited. In an attempt to improve such understanding, the NCI developed the Health Information National Trends Survey (HINTS), the first survey of its kind, to collect data on how Americans seek and use cancer information [17]. First administered in 2001, the survey will be conducted every two years to advance understanding and to bridge the gaps between the information patients want and need about cancer and the information they actually receive.

While the development of the NCI's HINTS instrument is an impressive step in facilitating awareness of the many ways in which individuals receive health information, perspectives on whether online health services have more positive or negative effects on patients remain varied. For example, researchers who conducted a study of Canadian oncologists and their patients determined that patients were three times more likely than oncologists to view Internet information as helpful to their ability to cope with their disease [18]. However, while a commonly held view on whether online health services are more helpful or hindering to patients does not exist, it is indisputable that accessing these services has some sort of impact on patients and their health outcomes.

As previously mentioned, researchers have discovered a positive connection between accessing information on specific health issues online and the psychological health of women with breast cancer. Of 188 women who were interviewed for a study examining the potential psychological benefits of using the Internet to access information related to breast health, 42% of the respondents used the Internet [12]. Using validated scales to measure social support and loneliness among the women with breast cancer, researchers determined that those individuals who used the Internet for medical information on issues surrounding breast health had more social support in their lives and experienced less loneliness than their counterparts who used the Internet for other purposes or who did not use the Internet at all [12].

Another study of women with breast cancer found that a 12-week, Internet-based social support group—*Bosom Buddies*—had a distinct impact on the health of the participants. Based on responses to six self-report scales and one group-report scale completed by the 72 women who participated in the study, researchers concluded that the *Bosom Buddies* support group effectively reduced participants' depression levels, perceived stress, and cancer-related trauma [16].

In an investigation of the relationship between the use of health information on the Internet and patient behavior and self-efficacy, results of a study conducted with individuals newly diagnosed with cancer indicated significant relationships among the variables. Specifically, researchers reported several notable findings: (1) 74% of Internet users compared to 54% of nonusers described their relationship with their physician as a partnership; (2) 81% of Internet users prepared a list of questions for their physician prior to their scheduled visit, while only 54% of nonusers prepared a question list; (3) Internet users (48%) asked six or more questions during the medical visit more often than nonusers (32%); and (4) when compared with nonusers, Internet users felt more confident in being able to participate in treatment decisions and to ask questions of their physicians [19].

Another outcome associated with utilization of online health services is the impact Internet health information has on the physician-patient relationship. In a telephone survey of a nationally representative sample of the American public, researchers found that 31% of the 3209 respondents had sought out health information on the Internet within the previous 12 months [20]. Overall, data from the study indicated that patients believe that online health information positively impacted their relationship with their physician [20]. Patients who perceived their physicians as having poor communication skills were typically the ones who reported that health information found on the Internet had a more negative effect on the physician-patient relationship [20].

While the aforementioned study does not relate solely to patients with cancer, findings from a similar study conducted exclusively with patients living with cancer echo the previous findings. In an attempt to examine Internet use among Australian oncology patients, researchers administered questionnaires to patients in two teaching hospitals in Sydney in 1999 and 2001. Data from both years suggested that information acquired from the Internet was perceived by patients as having either a positive or neutral influence on their relationship with their physician [21]. Notably, none of the 142 patients who completed the questionnaire during the 1999 study and a mere 3% of the 153 patients who participated in the 2001 survey indicated that online cancer information had a negative impact on the physician-patient relationship [21]. The impact of online health services on the physician-patient relationship may not be viewed as an outcome directly related to a patient's physical health. However, it is an important impact to address nonetheless as the quality of the physician-patient relationship has been shown to influence areas that *are* directly linked to a patient's health. Several studies have demonstrated a positive link between physician-patient communication, patient satisfaction, and positive health outcomes [4,22-24]. For example, after administering satisfaction surveys via telephone interviews with more than 230 adults who had seen a primary care physician within six months of the call, researchers determined that positive interaction and relational communication between physicians and patients significantly affected health outcomes such as compliance with medical treatment [23]. Consequently, the value of examining the impact of online health services on the physician-patient relationship should not be underestimated.

Most of the health outcomes mentioned here related to accessing online health services have been positive; however, negative effects have been reported as well. For example, 38% of 1050 physicians surveyed about their perceptions of the impact of Internet health information on the physician-patient relationship, health care, and workload indicated that clinical visits were less time efficient when patients brought information retrieved online [25]. Only 16% of the physicians surveyed believed that the effect of the patient bringing medical information retrieved online was beneficial [25]. Furthermore, an overwhelming 75% of the physicians noted that the online health information made no difference to the patient's health outcomes; 4% believed that the information was actually harmful to the outcomes [25].

Another study examined depression in people living with cancer using Internet and face-to-face support groups. Researchers found that patients with cancer who are more depressed, as measured by the Center for Epidemiologic Studies Depression Scale (CES-D) [26], use Internet support groups instead of face-to-face groups [27]. Considering that researchers have also found that people—not necessarily people with cancer, but people in general—become more depressed as they spend more time online [28], this finding may have further implications for the health outcomes of people living with cancer.

## Conclusion

While obvious benefits are associated with utilization of online health services among individuals living with cancer, such services are not infallible, as shown by several studies illustrating negative health outcomes that may be attributed to online health service use. By accessing accurate, reliable health-related information online, patients have the ability to equip themselves with information that enhances their understanding of and supplements the information they garner directly from their health care provider. However, online health information can often be confusing for patients to decipher, and, perhaps more importantly, it can often be conflicting or erroneous [14].

Notably, more than 70% of Internet users report that their treatment decisions are influenced by health information they find online [6]. For this reason, it is essential that investigation into the accuracy and dependability of online health information as well as the outcomes associated with utilization of online health services remains ongoing. Furthermore, as researchers begin to better understand the short-term impact online health services have on patients, they need to begin to address whether long-term effects exist as well.

## Abbreviations

CES-D: Center for Epidemiologic Studies Depression Scale
CIS: Cancer Information Service
HINTS: Health Information National Trends Survey
NCI: National Cancer Institute
